# Identifying enabling strategies for effective public dialogue in human embryo research

**DOI:** 10.1016/j.stemcr.2025.102498

**Published:** 2025-05-01

**Authors:** Matilda Beckett, Sarah Franklin, Peter J. Rugg-Gunn

**Affiliations:** 1 Epigenetics Programme, Babraham Institute, CB22 3AT Cambridge, UK; 2 Department of Sociology, University of Cambridge, CB2 1SB Cambridge, UK; 3 Loke Centre for Trophoblast Research, University of Cambridge, CB2 3EG Cambridge, UK; 4 Cambridge Stem Cell Institute, University of Cambridge, CB2 0AW Cambridge, UK

## Abstract

Public dialogue is crucial for understanding societal views on human embryo research, and the complexity and sensitivity of this topic require special considerations of how such dialogues are facilitated. Here, we identify enablers of effective dialogue, which can improve the design and delivery of engagement exercises related to embryo research.

## Introduction

**Figure 1:**
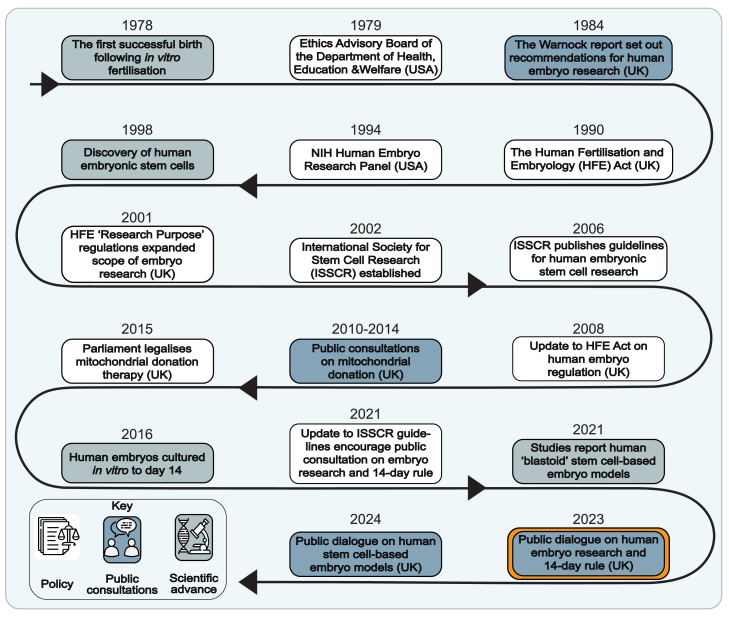
Timeline of key public consultations, policy changes, and selected scientific discoveries in human embryo research The public dialogue project relevant to this study is highlighted in orange.

Understanding how human embryos develop has long fascinated scientists and the public alike, and this area of research involves deep connections between science and society (Franklin and Jackson, 2024). Public opinion in the UK has shaped embryo research legislation since the Warnock Inquiry in 1984, which emphasized that embryo research laws should reflect moral and societal values. The inquiry led to the Human Fertilisation and Embryology (HFE) Act of 1990 and the establishment of the Human Fertilisation and Embryology Authority (HFEA) that regulates embryo research in the UK. Subsequent amendments in response to scientific and clinical advances over the past four decades have been substantially informed by public consultation and dialogue (Figure 1). Since 2000, the use of public dialogue has become an increasingly important component of UK science policy.

At the core of the HFEA governance model is the principle that clear red lines are essential for public support of controversial areas of scientific innovation. The 14-day limit established by the HFE Act permits human embryos to be cultured *in vitro* for up to 14 days after fertilization, but only subject to a strict licensing procedure bound by a rigorous code of practice. Following the passage of the HFE Act in 1990, the 14-day limit for human embryo research was adopted by many jurisdictions around the world. When conceived 40 years ago, the 14-day limit was largely theoretical, as it was not technically possible to culture embryos for more than a few days. Since 2016, however, new methods have enabled human embryos to be cultured for up to 14 days and non-human primate embryos for over 20 days. These discoveries have prompted discussion and review of the 14-day limit in many countries (Hyun et al., 2016).

The International Society for Stem Cell Research Guidelines for Stem Cell Research and Clinical Translation (version 1.0, May 2021) recommended that public engagement is an essential tool for understanding the societal and ethical considerations that would be raised if the 14-day limit were extended and to determine whether there is public support for such a change (Lovell-Badge et al., 2021). Several surveys have captured snapshots of public attitudes to extending the 14-day limit (Yui et al., 2023; 2024). In 2023, the UK Human Developmental Biology Initiative (HDBI) and Sciencewise commissioned an in-depth dialogue exercise to explore public views about human embryo research including the 14-day limit (hereafter, “the HDBI dialogue” [Hopkins et al., 2023] and summarized in Note S1). Public dialogue is a formal process during which members of the public interact with scientists and other stakeholders through a professionally structured format to deliberate on sensitive innovation and translational issues. Dialogues aim not to change opinions or reach consensus but to foster mutual learning between participants and are increasingly used to discuss ethically sensitive scientific topics (Nisbet and Scheufele, 2009). This form of engagement is recognized as being a particularly valuable approach to understand the reasons underpinning the views held by members of the public, which can inform the development of improved and more equitable research programs and policy (Sugarman et al., 2023). Given its recent emergence as a policy challenge, there are few examples of public dialogue specifically on the question of extending the 14-day rule, and therefore only limited understanding about whether special considerations might be required in terms of how such dialogue is facilitated. By using the HDBI dialogue as a case study, the analysis presented here is aimed to better understand what public dialogue means to both public and professional participants, to more clearly define the mechanisms that enable its success, and on this basis, to offer practical recommendations for implementing future public dialogue projects in this area.

## Defining successful public dialogue concerning human embryo research

The HDBI dialogue project involved 70 broadly representative members of the public who participated in an exploratory engagement exercise aimed not only to produce a snapshot of public views and perceptions but also to involve participants in a more extended conversation over several weeks. The project was designed and delivered by dialogue facilitators and public engagement professionals, with support from an oversight group including biologists and social scientists, bioethicists, science historians, legal specialists, regulators, and policymakers. The project was designed to adhere to established guidelines for public engagement exercises and was independently evaluated (URSUS Consulting). Although the participants were selected to represent a range of views, backgrounds, and ages, the dialogue project findings were intended to be indicative rather than representative. In addition to fostering and promoting mutual understanding of sensitive and complex topics, an important function of dialogue exercises is to help identify specific factors that may influence participants’ views and perceptions—some of which may be unanticipated and novel. This methodology is sometimes described as “factor finding” research and is especially useful for under-researched topics that are too new, multifaceted, or unfamiliar to fit well into convention poll, survey, or questionnaire methodologies. Indicative preliminary findings from such in-depth qualitative exercises can also be used to assess public views and opinions comparatively—both in the present and over time—and, where they are found to suggest a pattern of perception or behavior, the data from such exploratory exercises can then be used to more accurately design larger scale quantitative studies, such as polls or surveys.

Our study is focused on the methodologies used in such exercises and also on the question of how best to evaluate them. For the research reported here, we interviewed a small sample of 11 individuals who were involved in the HDBI dialogue project: three members of the public and eight contributors with different specialist roles (see Note S2). Although they are preliminary and indicative, rather than large-scale or representative, the findings from our study are of particular value and importance in helping to (1) illustrate how public engagement processes are understood by participants, (2) categorize the elements participants identify with more and less successful exercises of this kind, and thus (3) improve the evaluation criteria used to inform the design of similar future exercises.

**Figure 2:**
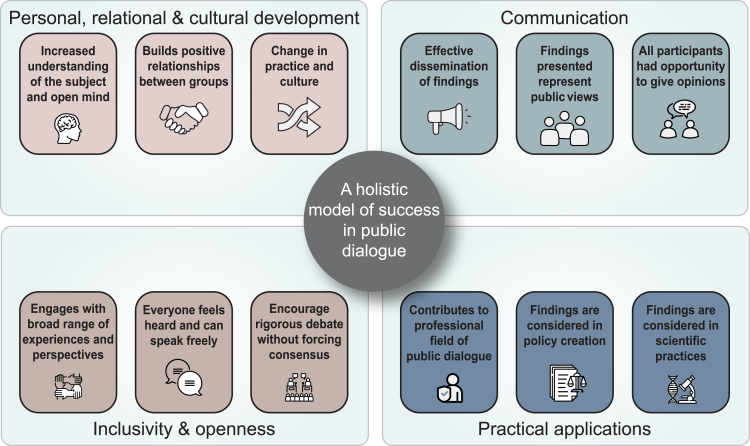
Holistic model of success in public dialogue, based on the data collected from our study See also Table S1.

To conduct our research, all of the HDBI dialogue participants we interviewed were asked to define what success in public dialogue meant to them, and these criteria were in turn analyzed with a view to identifying key elements that were perceived to contribute to more or less successful outcomes. A wide variety of answers were received (see Table S1), enabling us to propose an inclusive and comprehensive model of factors that are seen to enhance the success of public dialogue exercises. We sorted these key elements and factors into four broad categories: (1) personal, relational, and cultural development; (2) effective communication; (3) inclusivity and openness; and (4) practical application (Figure 2). Together, these four categories enabled us to provide a holistic account of the key elements seen to determine success in public dialogue.

## The role of “likely opposers”

Inclusivity and openness were strongly emphasized by all of the interviewees as a key element in successful public dialogue. A majority of interviewees agreed that the HDBI dialogue was strengthened by including public participants and specialists who were likely to oppose human embryo research and by initiating discussions about reasons to oppose human embryo research. While discussions in the dialogue often focused on opposition due to religious views, not all religions oppose such research, and there are non-religious reasons for opposition, which were also explored. The value of including opposing views was linked to another key factor, namely the possibility for personal and cultural development to emerge out of the dialogue process. Two public participants who identified themselves at the start of the project as “likely opposers” reported appreciating diverse perspectives and the freedom to express their views—but also that their own views changed as a result of the openness of the dialogue. As an example, Public Member 1, from a Catholic background, described how their opinions evolved and their “mind was changed” by the exercise:

When I first read it all at the beginning, I was probably against it because I was thinking, no, that’s an embryo, what if it feels pain? What if it has a soul? No, I couldn’t really agree with that. But how my mind was changed, information is power, isn’t it?—Public Member 1

Whether or not their minds were changed, participants also valued their exposure to a range of views in terms of gaining deeper insights into the views of others. Public Member 3, whose views were shaped by living with a family member with a developmental condition as well as by their Christian faith, did not change their overall opinion but enjoyed gaining a better understanding of opposing views, for example through hearing personal stories from individuals who had had children through assisted reproduction:

When you actually spoke to and heard stories from people and how the drive to have children really affects people, from that I can see a different side of what people are doing in terms of embryo research.—Public Member 3

Professional Member 3 noted that including religious perspectives challenged preconceived notions and improved methodological robustness.

## Informal communication, downtime, and reflection

In addition to content, interviewees in our study also drew attention to formal aspects of the dialogue exercise that were seen to contribute to its success. These included the amount and pacing of communication, both of which were seen to play an important role in enabling the kind of reflection that allowed participants to “see different sides,” as mentioned earlier. Some participants emphasized the importance of informal conversation and reflection periods as a way to build relationships, enhance understanding, and incorporate additional viewpoints. Varying the format of communication also mattered. For example, Professional Member 1 mentioned the value of one-on-one conversations during coffee breaks at in-person workshops during which participants could ask clarifying questions, improving both their understanding of the topic and relationship-building within the group.

While these face-to-face experiences were absent from online sessions, the initial webinars were praised by interviewees for including pauses and downtime. Both public and professional dialogue participants emphasized the importance of including time to reflect in the webinar.

I particularly liked the webinar because there was a lot of time to kind of digest the materials and be able to kind of reflect and have time to think.—Public Member 2

Access to multiple online options was another aspect of the webinar praised for enabling space to explore related themes as well as time to reflect on their importance. A supplementary resources platform that was frequently used by participants before and between sessions offered the opportunity to complete activities, review workshop recordings, access news articles, submit questions, and engage in discussion boards.

The duration of the dialogue exercise also allowed for discussion of topics outside of formal sessions, and this too was viewed as advantageous. In keeping with Sciencewise’s guidelines, participants were neither discouraged nor encouraged to discuss dialogue topics with family and friends. Public Member 1 engaged their family in these conversations, which in turn was described as having influenced their own opinion about human embryo research—again reinforcing the theme of time to reflect, explore, and understand different sides of the issue, but also, in this case, further supporting the importance of casual discussion to the process of evaluating complex information.

Taken together, incorporating break times, reflection periods, access to supplementary resources, and allowing space for informal discussions outside the group over the course of the exercise were all identified as helpful components of the HDBI dialogue by public and professional members. These elements were valued as means to increase understanding, improve relationships, reflect on “different sides”, and “test” ideas, to arrive at more informed, nuanced, and confident views on a highly technical topic that many of the public participants had never previously encountered and knew little about.

## High-quality facilitation

The role of facilitators in delivering a successful dialogue and engagement experience for public and professional participants is clearly a paramount concern in exercises of this kind. Effective facilitation is crucial for fostering respectful, participant-focused, and informed but impartial discussion. Five of 11 interviewees explicitly highlighted the skill and experience of facilitators as a major contributor to the success of the HDBI dialogue.

In addition to logistical and practical guidance, facilitators also provided emotional support and were praised for having handled emotionally charged topics well.

The facilitators clearly had a lot of experience in dealing with these sorts of elusive personal aspects of the issues that may come up and I thought they handled it very well.—Professional Member 4

Facilitators also acted as moderators to help support and guide group discussion. This is a role for which people are trained, and the UK has a larger number of such professionals than most countries due to the importance of engagement and dialogue to UK science policy (Burchell et al., 2009). Professional participants, who relied on the facilitators to decide when to interject with factual information without overtaking the conversation, appreciated the assistance this offered to dialogue.

I really relied on the facilitators to judge when to bring me in or not, because for the most part I didn’t want to say anything because […] I think as an expert the risk is that you can overtake a conversation.—Professional Member 2

Facilitators also helped to create a safe and inclusive environment by inviting quieter participants to share their thoughts and tactfully preventing any individual group members from dominating discussion. They also moved the discussion forward and helped to cover as much ground as possible by raising questions, encouraging interaction, and integrating viewpoints from breakout groups. This role was appreciated by participants for several reasons, including the ability to hear and reflect on opposing points of view in a well-structured environment that enabled all voices to be heard.

I would speak about my opinion for a bit and they would say “we’re going to come back to you, but we’re going to have to move on and get everybody’s point of view” […] At the end we would have a chance to disagree or add to other people’s thoughts or change our initial opinions […] You mightn’t think of the other implications of what you’ve said until someone steps up and says “but hold on a minute have you thought about this”—Public Member 1

## Online and in-person dialogues

There is a limited literature on the pros and cons of online versus face-to-face engagement and dialogue, despite the significant increase in the use of online webinars in the post-COVID period. Views about the comparative value of online versus in-person dialogue were a common topic of discussion among participants, with interviewees identifying a range of benefits and limitations to both formats (see Table S2). Although in a preliminary manner, our findings pointed to several key themes in this area that could inform future research and dialogue project design. For example, while virtual dialogues are often perceived to cut venue and travel costs, Professional Members 5 and 8 questioned if they are truly cheaper, as expensive virtual tools are needed for high-quality, interactive experiences.

Commissioners tend to think about online dialogue being cheaper and quicker. It’s not really [either of these] because you have to design it just as carefully, if not more carefully, to make it work well online.—Professional Member 8

The strongest argument in favor of virtual dialogue was its accessibility. Professional Member 3 noted that online formats can make dialogues accessible to people with constraining home and work situations. This view was echoed by multiple interviewees.

Having a virtual aspect to the sessions also enables different people to participate because there will be people who won't be able to physically come to sessions, who would be able to take part virtually.—Professional Member 4

Additionally, Public Members 1 and 2 stated that online dialogues felt less overwhelming. The online platform allowed them to consider their thoughts and opinions before speaking, reducing the initial intensity of being introduced to new concepts as well as new people in a face-to-face setting. Despite these benefits, interviewees highlighted several limitations of online sessions, including the lack of spontaneity and flow and the related need for more direct prompts from facilitators, whereas in-person dialogues allowed participants to converse more freely. At the same time, greater facilitation and less spontaneity, as well as the option of self-directed interaction with online materials, were also seen to have their own positive effects on success. Online discussions were praised for better management and fewer interruptions, with chat functions allowing participants to introduce new ideas without speaking over each other. Conversely, the relational benefits of more animated conversation in a live group setting were often reduced, or lost entirely, in online activities, and there was an accompanying concern that participants might be distracted by their home or work environments. Our findings suggest that the greatest benefit may lie in hybrid approaches, but that timing also matters. Less intimidating introductory sessions can enable participants to become comfortable with each other, with the benefits of socialization and flowing discussion in the in-person discursive workshops developing subsequently.

## What is neutrality in public dialogue?

A key objective of the HDBI dialogue project was to inform, explore, and develop participant viewpoints without imposing any particular consensus or bias. In general, public engagement and dialogue projects are neither designed to reach definitive conclusions nor to establish an “objectively neutral” middle ground. They are instead intended to create a context in which the exchange of views can be mutually illuminating to all involved *because* they avoid steering the group toward any foregone conclusion or determining the “right” answer. While interviewees generally agreed that the dialogue was successfully structured to avoid these outcomes, there were different views as to whether neutrality was achieved, or indeed, if it is ever truly possible. The HDBI dialogue featured a broad oversight group with representation from diverse fields including medical ethics, developmental biology, sociology, history, fertility advocacy, public dialogue, policy, medical history, and law. This diverse input combined with skilled facilitation was praised in retrospective assessments of the project by one participant as “a good mix”:

This was a very good oversight group, very engaged, very excited about the work […] they came from a lot of different fields, a good mix, and they played a very active role in framing the dialogue, but also in contributing as specialists and then disseminating the report and sharing the findings more widely at the end.—Professional Member 8

Careful delivery of dialogue was crucial for this blend of perspectives to generate a lively and challenging exchange that could push participants to “test” their own viewpoints, and disagree, but also ask questions and become better informed. Such a format requires careful balances and a degree of restraint. It is important, for example, that scientific literacy is not a barrier to participation. Scientific specialists acknowledged this tension: they wanted to correct biological details in order to be “fully accurate” but also knew they needed to act primarily as observers. This approach challenged them but ensured participants remained central to the dialogue.

However, some participants felt that a complete representation of diverse views was not fully achieved. Public Member 3, who opposed embryo research, felt their views were acknowledged but given less weight compared to those in favor. Public Member 2 felt that the dialogue was not biased in any direction but noted that it contained limited voices opposing human embryo research or extension to the 14-day limit.

What I would say is that it would have been good to hear more voices from people who may be against the extension […] I didn’t really hear that much in terms of […] people who would have been in disagreement about the extension of the fourteen-day window.—Public Member 2

Despite these concerns, interviewees cited participants’ development of independent ideas, such as reversible regulation and lay summaries of research, as evidence of freedom from bias.

They had lots of opportunities to identify both the downsides and the potential benefits of the research and … how they wanted to see research governed in the future … they came up with a lot of their own suggestions, particularly on the governance side which weren’t being proposed or suggested by the researchers at all.—Professional Member 8

From the interviews conducted, it thus appears that while the dialogue was perceived to be as inclusive and unconstrained as possible, achieving a full spectrum of views may be unfeasible given the small size of the group and the short time frame of the project. One professional member suggested that, even if the organizers genuinely had no agenda, participants may perceive one, although they added that they felt the public participants would still be confident in their own views. Another professional member noted that since the dialogue is shaped by both organizers and participants, who all bring their own lived experiences to bear on their evaluation of the questions at hand, nobody involved can be fully objective and neutral. This interplay helps balance any perceived agendas and might approximate a rough neutrality overall.

My experience is that there was probably enough intransigence on both sides that perhaps something kind of averaged out in the middle.—Professional Member 3

## Discussion

Public consultation on human embryo research has continued to expand and evolve for over 40 years. Since 2000, the importance of public engagement and dialogue concerning innovative and controversial scientific developments has become official UK science policy. As a result, the UK has a comparatively well-developed public engagement and dialogue skill base, with several groups having emerged to provide this service, including some, such as Sciencewise, that are partially funded from within government. As methodologies for conducting dialogue and engagement exercises continue to evolve and means of evaluating such exercises also develop further, studies such as ours can help provide empirical evidence and insight into considerations and strategies for effective public dialogue on sensitive topics such as human embryo research.

Our study looked at a novel dialogue exercise from the point of view of trying to identify factors that influenced public and professional participants’ views of successful outcomes. Based on in-depth data collected from a subset of dialogue participants through interviews, we concluded that four key groups of criteria emerged as “priority factors” determining success in this particular case study. These are schematically outlined in Figure 2, and in our discussion, we have selectively illustrated these broad groupings using examples of insights gained from participants. As these examples show, the ability for participants to learn, engage, and increase their own understanding of a complex topic benefited from a number of specific dialogue features. In addition to skilled facilitation, participants valued a mixed set of opportunities to improve their understandings and refine their views. These included the value of face-to-face interaction combined with independent learning; the benefits of online independent and group work combined with live, in person sessions; and the ability to hear from professional specialists dealing largely in facts and also from other members of the public who expressed different viewpoints. We also found that good facilitation, time to reflect, and casual conversations with family and friends provided important opportunities to explore, test, refine, and change their views. To ensure a “good mix” of views and materials, the HDBI dialogue provided multiple resources and opportunities to participants that included reasons to oppose as well as support extension of the 14-day limit and/or human embryo research in general. These included discussion prompts, pre-recorded videos, and specialists who presented arguments for opposition. This model of constructive communication, informed debate, and friendly curiosity ensured a dialogue that was robust and reflective of societal values, providing a hopeful example for building relationships among different communities. Future dialogues could learn from models like the AAAS Dialogue on Science, Ethics, and Religion, which focuses on relationship-building between scientists and faith communities, engaging with experts on how to navigate the historical events that have built such tension and training facilitators to converse about complex topics such as the pro-life movement (AAAS, 2020; O’Malley et al., 2021).

Public dialogue is integral to many research fields, such as mitochondrial donation, germline gene editing, and artificial intelligence. In germline gene editing, clear goals for public engagement have been identified, including fostering deliberation on “what-if” scenarios, exploring value-based agreements and disagreements, involving diverse perspectives, and informing socially aligned policymaking (Geuverink et al., 2024). In common with human embryo research, literature from germline gene editing emphasizes the need to embed public dialogue into the scientific research infrastructure to enhance its credibility (Scheufele et al., 2021). In the field of AI, ethical concerns echo those in human embryo research, particularly around respect for humanity, unforeseen future consequences, and informed consent (Pickering, 2021). Challenges to public engagement include how to navigate the complexities and uncertainties of the topics, promote transparency, counter misinformation, and facilitate inclusive dialogue (Adams and Burall, 2019). A systematic review of public dialogues on ethically contentious subjects could further clarify how generalizable findings are across fields and help define what methodologies are most effective at facilitating dialogue on such topics.

## Acknowledgments

We thank the individuals who participated in our study. We are grateful to Naomi Clements-Brod and Michael Norman for their comments on the manuscript. P.J.R.-G.’s group is supported by grants from the 10.13039/501100000268BBSRC (BBS/E/B/000C0522), 10.13039/501100000265MRC (MR/T011769/1 and MR/V02969X/1), and 10.13039/100004440Wellcome (215116/Z/18/Z and 225839/Z/22/Z).

## Author contributions

Conceptualization, S.F. and P.J.R.-G.; methodology, M.B., S.F., and P.J.R.-G.; investigation, M.B.; data curation, M.B.; analysis, M.B.; writing, M.B., S.F., and P.J.R.-G.; project administration, P.J.R.-G.; funding acquisition, S.F. and P.J.R.-G.; supervision, S.F. and P.J.R.-G.

## Declaration of interests

The authors declare no competing interests.

## Supplemental information

Document S1. Tables S1 and S2 and Notes S1 and S2

Document S2. Article plus supplemental information
