# Supplementary material for: Identifying enabling strategies for effective public dialogue in human embryo research
Source: Stem Cell Reports. 2025 May 1;20(5):102498. doi: 10.1016/j.stemcr.2025.102498 (PMC12143149; doi:10.1016/j.stemcr.2025.102498)
Supplement: Document S1. Tables S1 and S2 and Notes S1 and S2 [file mmc1.pdf]

Stem Cell Reports, Volume *20*

Supplemental Information

Identifying enabling strategies for effective public dialogue in human embryo research

Matilda Beckett, Sarah Franklin, and Peter J. Rugg-Gunn

Table S1. Definitions of success for public dialogue, related to Figure 2.

Interviewee	Definition of success
**Public Member 1**	Create rigorous discussions that include diverse viewpoints without forcing consensus. Collect public perspectives and feed them into policy or practice.
**Public Member 2**	Ensure individuals leave with a more open mind and increased knowledge, with opportunities to provide personal feedback.
**Public Member 3**	Enable organisers and participants to listen to and engage with a diverse range of life experiences and viewpoints.
**Professional Member 1**	Focus on making sure everyone involved feels heard and able to express their opinions.
**Professional Member 2**	Capture public discussions to crystallise opinions and attitudes that may inform policy.
**Professional Member 3**	Catalyse changes in practice and of culture.
**Professional Member 4**	Collect findings that accurately represent public views and can be used for policy development. Foster positive relationships between stakeholder groups.
**Professional Member 5**	Ensure participants feel more engaged with the topic, and collect findings useful for policy, practice, or future dialogues.
**Professional Member 6**	Advance the field of public dialogue. Enhance participants' scientific knowledge and understanding of alternative viewpoints. Provide commissioners with insights into public perspectives.
**Professional Member 7**	Facilitate meaningful conversations between participants and scientists, and accurately capture these discussions.
**Professional Member 8**	Achieve pre-set objectives of design, policy impact and contribution to the professional field of public dialogue. Effectively disseminate findings.

Table S2. Comparison of Online and In-Person Public Dialogue, related to section on 'Online and in-person dialogues'.

	In-person dialogue	Online dialogue
**Cost**	Higher costs due to venue and travel expenses.	No venue or travel costs.
		Creating engaging online experiences can be costly.
**Accessibility**	Accessibility and convenience limited by geography, work hours, children, disabilities, carer duties, etc.	Allows participation from various locations with reduced travel and timing constraints. Online environment may be less overwhelming for some participants.
**Discussion and debate**	Stronger rapport through face-to-face interactions. Immediate answers to questions available. Increased social bonding and casual interaction.	Participants may feel less social pressure and provide more honest, unfiltered opinions.
	Potential for social pressure to reduce the openness of viewpoints expressed.	Harder to build rapport and relational goals. Discussions may be less memorable over time.
**Facilitation**	Easier to facilitate discussions when able to see body language. Increased spontaneity of discussion between participants.	Easier to manage speaking order and reduce interruptions. Chat function allows for non-interruptive additional points.
	Harder to manage interruptions and side-conversations.	Conversations tended to flow between facilitator and participant rather than between participants. Harder to respond to participants' non-verbal cues.
**External distractions**	Reduced likelihood of distractions due to being in a dialogue-specific location.	Higher risk of participants being distracted by events in their home or at work.

Note S1 – A summary of the HDBI Public Dialogue, related to the introduction.

The HDBI Public Dialogue project sought to engage a diverse group of the UK public to deliberate on early human embryo research with a specific focus on the 14-day rule. Co-funded and commissioned by the Human Developmental Biology Initiative and UK Research and Innovation Sciencewise, and delivered by Hopkins Van Mil, this foundational project aimed to hear and discuss public hopes and concerns for this area of research. This was a qualitative study to understand why views are held and what is important to individuals.

The project used targeted recruitment to obtain a range of levels of awareness of early human embryo research and regulation, and a mix of those who support and oppose this research, enrolling 70 members of the public from across the UK. To support the project delivery team, an Oversight Group was formed of 23 members comprising biologists and social scientists, third sector organisations and those involved in embryo research policy. Over the course of the project, 28 specialists provided input that covered biological sciences (12 individuals), regulation and legislation (4), philosophy and ethics (7), medicine (2) and patient views (3).

The project started with a pilot group of 9 members of the public, followed by a second phase of the project with 19 individuals who have lived experiences of developmental conditions, fertility treatment, or recurrent miscarriage. The process for the pilot and lived-experiences groups involved a webinar and three online workshops. The next stage of the project was with two general groups, each of 21 members of the public. For these groups, the last two workshops were held in-person. Presentations from specialists, lived experience films and infographics were used as stimulus materials for the discussions. An online platform, available between sessions, enabled participants to complete activities, watch workshop recordings, access news articles, submit questions, and participate in discussion boards.

Public participants of the project showed high engagement and fascination about early human embryo research. The current knowledge gaps in embryo research were easily grasped, particularly the limited information available on how embryos develop between day 7 and day 28. Ethical, moral and religious considerations featured throughout the discussions. There was a high level of confidence in the current regulatory and legislative structures that surround early human embryo research. Many participants voiced hope for further research particularly where there is opportunity for improvement in human health.

Participants' concerns for the future included worries related to the detection of certain health conditions or disabilities, which could then lead to the elimination of those conditions and without society's consent. There were also worries about genetic engineering, and whether embryo research would enable the creation of 'perfect' individuals. Concerns were raised about who was funding embryo research, and the possibility of inequitable access to future treatments.

Many participants supported some form of extension to the 14-day rule provided it aligns with societal expectations regarding respect for the embryo and the research remains rigorously regulated. Common reasons provided why individuals would support an extension to the current limit included the potential of the research to lead to improvements in IVF success rates, reduce the incidence of miscarriage, and to better understand, treat or prevent serious health conditions. Views differed between individuals on how the 14-day rule should change. Some believed the change should occur gradually, in small increments, with regular reviews. Others argued that extending the limit to 28 days should be considered, given the potential new benefits the research could offer, particularly during the 14- to 28-day developmental period. There was a strong interest to involve the public in future decision making on this area of research and that these processes are transparent.

An independent evaluation report concluded that the Public Dialogue project was well-designed, efficiently delivered, and successfully met all of the original objectives. The evaluation noted that conditions were successfully created to allow participants to share views and discuss sensitive topics. The mix of stimulus materials, ample time for small group deliberation both online and in person, and substantial involvement of specialists contributed to the effective delivery and impact of the project.

Note S2 – Participant recruitment and interviews, related to the section on 'Defining successful public dialogue concerning human embryo research'.

Approval to conduct the interview-based research was obtained from the University of Cambridge Sociology Ethics and Risk Assessment for Research Committee. Eleven individuals who participated in the HDBI dialogue were interviewed. These individuals represent a broad range of positions within the dialogue: members of the public who participated in the exercise, professionals who oversaw design and delivery of the project, specialists who provided sessions for the participants during the exercise, and project evaluators. Interviews were conducted either in-person or online, and were semi-structured with an interview guide. Questions explored participants' experiences with the HDBI public dialogue, their views on different methodologies, and their personal definitions of success for public dialogues, among other topics. Interview recordings were transcribed using Microsoft Word Speech to Text and edited for accuracy. All identifying information was removed from transcripts and audio files were deleted after use. Analysis was indicative, formed by close reading and notetaking.

<u>1: Contact message</u>

Dear [Recipient's Name],

I hope this email finds you well. My name is Matilda Beckett, and I am reaching out to you regarding a study on high-quality public dialogue in early human embryo research. Your association with the HDBI public dialogue on this topic makes your perspective invaluable to our research, and I would like to invite you to participate in a 30-minute to 1-hour interview.

The purpose of my study is to gain insights into what constitutes high-quality public dialogue in early human embryo research and to explore why it matters. Your unique experiences and expertise would contribute significantly to the depth and richness of the findings.

Interviews can be conducted either online at a time convenient for you or, if you are based in Cambridge, in person. I understand the importance of your time, and I assure you that the interview will be structured efficiently while allowing time and space to explore interesting themes.

Your participation will contribute to advancing our understanding of public dialogue in this critical field.

If you are willing to participate, please reach out to me at [redacted].

I appreciate your time and consideration in contributing to this research endeavour.

Warm regards,

Matilda Beckett

MPhil Reproduction and Embryogenesis

University of Cambridge

2: Consent Form

Title of Project: What does High Quality Public Dialogue in Early Human Embryo Research Look Like and Why does it Matter?

Researcher: Matilda Beckett – MPhil Reproduction and Embryogenesis

About the Project: This project seeks to investigate the use of dialogue-based methods to understand public opinions on human embryo research. The project utilises the recent HDBI Public Dialogue on Early Human Embryo Research as a case study. By interviewing scientists, public engagement professionals and participants of the dialogue, the project aims to identify which aspects of public dialogue-based methods are most effective and which should or should not be taken forward to future engagement activities. The project also hopes to factor find by uncovering factors that influenced decision making for participants and researchers throughout the public dialogue process. The expected outcomes are i) a deepened understanding of what high-quality public engagement might look like for the field of early human embryo research, ii) a rationale as to why the dialogue aspect of public engagement is important and iii) anticipation of future directions for dialogue involving early human embryo research and similar topics.

During the interview you will be asked questions about your experience of the HDBI public dialogue activity, your role in the public dialogue and hypothetical questions about the future of public dialogue. Participation is voluntary and you have the right to withdraw at any time with no consequence by contacting the email provided on this form. Interviews will be audio recorded on a secure device. Personal identifying information will be removed, and transcripts will be analysed for overall themes. Some quotes may be used in the project write-up. Interviews will be carried out in-person and online. Some interviews may also be carried out using questionnaires.

Your data will be processed according to the guidance provided by the University of Cambridge regulations on secure storage and use of participants' data. Anonymised transcripts will not be made available for use by researchers other than those within my supervisors' groups at the University of Cambridge and the Babraham Institute. You may consent or refuse consent to future use of interview data below.

If you would like more information about the data storage and participant anonymisation methodology for this project, please feel free to contact me at [redacted].

The University's regulations on the secure storage and use of participants' data may be found at https://www.information-compliance.admin.cam.ac.uk/dataprotection/research-participant-data

1. I confirm that I have understood the purpose of the project and my participation in it, and I have had the opportunity to ask questions. ☐

2. I understand that my participation is voluntary and that I am free to withdraw at any time without giving any reason. ☐

3. I understand that my responses will be anonymised and used only for academic research. ☐

4. I understand that my interview may be recorded. ☐

5. I agree to take part in the above project. ☐

6. I consent for anonymised data collected through these interviews to be stored securely and potentially used in follow up studies. Yes ☐ No☐

Signatures

Participant name signature and date

Researcher name signature and date

3: Interview Guide

Key	
**Aim to ask to everyone**	*
**Of specific importance to professional members**	**
**Of specific importance to public members**	***

Can you tell me what your role was in this public dialogue project and how you got involved?	*
What was your experience of public dialogue before this?	*
How would you define public dialogue? What is your understanding of the difference between public dialogue and public outreach?	*
What activities and processes did participants/you go through in the HDBI public dialogue?	*
How can "success" be defined for a public dialogue activity?	*
Are there any aspects of public dialogue that you think make the dialogue activity more or less successful?	*
Do you think the HDBI Public Dialogue on Early Human Embryo Research was a success?	*
What are some examples of what went well in the HDBI public dialogue? Was this related to any particular people or activities?	*
What are some examples of aspects that either didn’t go well or could be improved?	*
How were discussions between participant groups facilitated?	*
Do any particular workshops, speakers, or activities stand out to you as particularly vital, decisive, or influential?	*
One of the aims of public dialogue is to encourage participants to come to conclusions unencumbered by the conscious/unconscious goals of the project researchers. Do you feel like this worked?	*
Considering specifically the aspects of the dialogue relating to stem cell-based embryo models, were there any particular strengths or limitations to this part of the dialogue which come to mind?	*

Do you think that in future dialogues it would be possible to run dialogues completely remotely? Were there any benefits to online versus in-person sessions?	*
I've experienced opinions in my cohort that the public is not interested in embryo research and is not well-informed enough to be involved. What would your response to this opinion be?	*
During the HDBI dialogue, did there appear to be any "tipping points" for participants/you changing or steadying opinions? What were they? Were these associated with any tasks or speakers?	*
In terms of translating public dialogue results into policy implications, how do you envision this going forward?	*
Was there anything that really surprised you during the public dialogue process, anything you weren't necessarily expecting?	*
Is there anything else that you think I might want to know that I've not directly asked about?	*
Considering the different groups of participants from the northern, southern, and lived experience groups, did you observe any differences in the flexibility of their opinions, or did they all seem equally plastic?	**
Do you think it makes sense to have a separate lived experience group? If so, why?	**
What do you feel are the main limitations of public dialogue overall?	**
Did you come into the dialogue feeling like you already knew what your opinions would be? Do you think other participants had the same/different levels of plasticity?	***
Now that you have been involved in two relatively lengthy public dialogues, would you still consider yourself a layperson on the topic of human embryo research?	***
