# Supplementary material for: Identifying enabling strategies for effective public dialogue in human embryo research
Source: Stem Cell Reports. 2025 May 1;20(5):102498. doi: 10.1016/j.stemcr.2025.102498 (PMC12143149; doi:10.1016/j.stemcr.2025.102498)
Supplement: Document S2. Article plus supplemental information [file mmc2.pdf]

Stem Cell Reports
Forum
OPEN ACCESS

Identifying enabling strategies for effective public dialogue in human embryo research

Matilda Beckett,[1,2] Sarah Franklin,[2,5] and Peter J. Rugg-Gunn[1,3,4,5,*]
[1]Epigenetics Programme, Babraham Institute, CB22 3AT Cambridge, UK
[2]Department of Sociology, University of Cambridge, CB2 1SB Cambridge, UK
[3]Loke Centre for Trophoblast Research, University of Cambridge, CB2 3EG Cambridge, UK
[4]Cambridge Stem Cell Institute, University of Cambridge, CB2 0AW Cambridge, UK
[5]Senior author
*Correspondence: peter.rugg-gunn@babraham.ac.uk
https://doi.org/10.1016/j.stemcr.2025.102498

Public dialogue is crucial for understanding societal views on human embryo research, and the complexity and sensitivity of this topic require special considerations of how such dialogues are facilitated. Here, we identify enablers of effective dialogue, which can improve the design and delivery of engagement exercises related to embryo research.

INTRODUCTION

Understanding how human embryos develop has long fascinated scientists and the public alike, and this area of research involves deep connections between science and society (Franklin and Jackson, 2024). Public opinion in the UK has shaped embryo research legislation since the Warnock Inquiry in 1984, which emphasized that embryo research laws should reflect moral and societal values. The inquiry led to the Human Fertilisation and Embryology (HFE) Act of 1990 and the establishment of the Human Fertilisation and Embryology Authority (HFEA) that regulates embryo research in the UK. Subsequent amendments in response to scientific and clinical advances over the past four decades have been substantially informed by public consultation and dialogue (Figure 1). Since 2000, the use of public dialogue has become an increasingly important component of UK science policy.

At the core of the HFEA governance model is the principle that clear red lines are essential for public support of controversial areas of scientific innovation. The 14-day limit established by the HFE Act permits human embryos to be cultured *in vitro* for up to 14 days after fertilization, but only subject to a strict licensing procedure bound by a rigorous code of practice. Following the passage of the HFE Act in 1990, the 14-day limit for human embryo research was adopted by many jurisdictions around the world. When conceived 40 years ago, the 14-day limit was largely theoretical, as it was not technically possible to culture embryos for more than a few days. Since 2016, however, new methods have enabled human embryos to be cultured for up to 14 days and non-human primate embryos for over 20 days. These discoveries have prompted discussion and review of the 14-day limit in many countries (Hyun et al., 2016).

The International Society for Stem Cell Research Guidelines for Stem Cell Research and Clinical Translation (version 1.0, May 2021) recommended that public engagement is an essential tool for understanding the societal and ethical considerations that would be raised if the 14-day limit were extended and to determine whether there is public support for such a change (Lovell-Badge et al., 2021). Several surveys have captured snapshots of public attitudes to extending the 14-day limit (Yui et al., 2023; 2024). In 2023, the UK Human Developmental Biology Initiative (HDBI) and Sciencewise commissioned an in-depth dialogue exercise to explore public views about human embryo research including the 14-day limit (hereafter, "the HDBI dialogue" [Hopkins et al., 2023] and summarized in Note S1). Public dialogue is a formal process during which members of the public interact with scientists and other stakeholders through a professionally structured format to deliberate on sensitive innovation and translational issues. Dialogues aim not to change opinions or reach consensus but to foster mutual learning between participants and are increasingly used to discuss ethically sensitive scientific topics (Nisbet and Scheufele, 2009). This form of engagement is recognized as being a particularly valuable approach to understand the reasons underpinning the views held by members of the public, which can inform the development of improved and more equitable research programs and policy (Sugarman et al., 2023). Given its recent emergence as a policy challenge, there are few examples of public dialogue specifically on the question of extending the 14-day rule, and therefore only limited understanding about whether special considerations might be required in terms of how such dialogue is facilitated. By using the HDBI dialogue as a case study, the analysis presented here is aimed to better understand what public dialogue means to both public and professional participants, to more clearly define the mechanisms that enable its success, and on this basis, to offer practical recommendations for

© 2025 The Authors.
Published by Elsevier Inc. on behalf of International Society for Stem Cell Research.
This is an open access article under the CC BY license (http://creativecommons.org/licenses/by/4.0/).

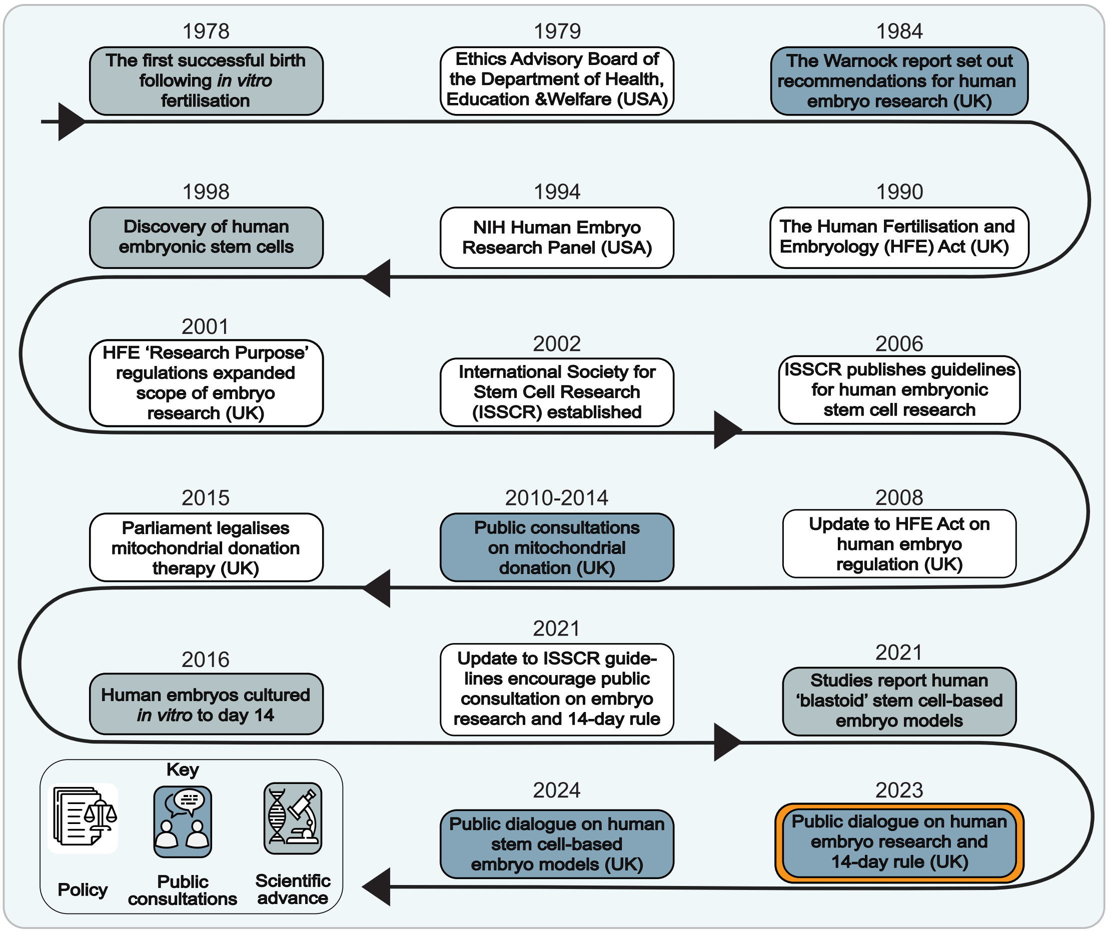

Figure 1. Timeline of key public consultations, policy changes, and selected scientific discoveries in human embryo research
The public dialogue project relevant to this study is highlighted in orange.

implementing future public dialogue projects in this area.

DEFINING SUCCESSFUL PUBLIC DIALOGUE CONCERNING HUMAN EMBRYO RESEARCH

The HDBI dialogue project involved 70 broadly representative members of the public who participated in an exploratory engagement exercise aimed not only to produce a snapshot of public views and perceptions but also to involve participants in a more extended conversation over several weeks. The project was designed and delivered by dialogue facilitators and public engagement professionals, with support from an oversight group including biologists and social scientists, bioethicists, science historians, legal specialists, regulators, and policymakers. The project was designed to adhere to established guidelines for public engagement exercises and was independently evaluated (URSUS Consulting). Although the participants were selected to represent a range of views, backgrounds, and ages, the dialogue project findings were intended to be indicative rather than representative. In addition to fostering and promoting mutual understanding of sensitive and complex topics, an important function of dialogue exercises is to help identify specific factors that may influence participants' views and perceptions—some of which may be unanticipated and novel. This methodology is sometimes described as "factor finding" research and is especially useful for under-researched topics that are too new, multifaceted, or unfamiliar to fit well into convention poll, survey, or questionnaire methodologies. Indicative preliminary findings from such in-depth qualitative exercises can also be used to assess public views and opinions comparatively—both in the present and over time—and, where they are found to suggest a pattern of perception or behavior, the data from such exploratory exercises can then be used to more accurately

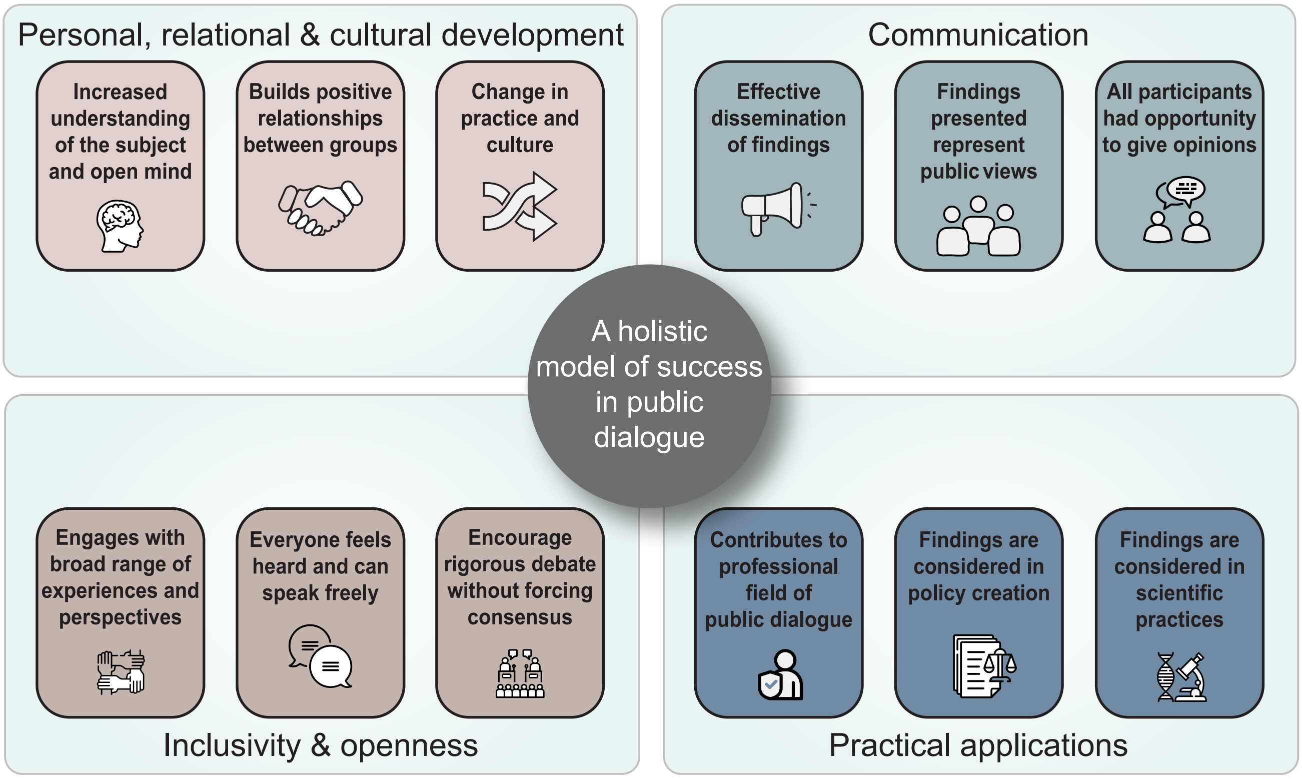

Figure 2. Holistic model of success in public dialogue, based on the data collected from our study
See also Table S1.

design larger scale quantitative studies, such as polls or surveys.

Our study is focused on the methodologies used in such exercises and also on the question of how best to evaluate them. For the research reported here, we interviewed a small sample of 11 individuals who were involved in the HDBI dialogue project: three members of the public and eight contributors with different specialist roles (see Note S2). Although they are preliminary and indicative, rather than large-scale or representative, the findings from our study are of particular value and importance in helping to (1) illustrate how public engagement processes are understood by participants, (2) categorize the elements participants identify with more and less successful exercises of this kind, and thus (3) improve the evaluation criteria used to inform the design of similar future exercises.

To conduct our research, all of the HDBI dialogue participants we interviewed were asked to define what success in public dialogue meant to them, and these criteria were in turn analyzed with a view to identifying key elements that were perceived to contribute to more or less successful outcomes. A wide variety of answers were received (see Table S1), enabling us to propose an inclusive and comprehensive model of factors that are seen to enhance the success of public dialogue exercises. We sorted these key elements and factors into four broad categories: (1) personal, relational, and cultural development; (2) effective communication; (3) inclusivity and openness; and (4) practical application (Figure 2). Together, these four categories enabled us to provide a holistic account of the key elements seen to determine success in public dialogue.

THE ROLE OF “LIKELY OPPOSERS”

Inclusivity and openness were strongly emphasized by all of the interviewees as a key element in successful public dialogue. A majority of interviewees agreed that the HDBI dialogue was strengthened by including public participants and specialists who were likely to oppose human embryo research and by initiating discussions about reasons to oppose human embryo research. While discussions in the dialogue often focused on opposition due to religious views, not all religions oppose such research, and there are non-religious reasons for opposition, which were also explored. The value of including opposing views was linked to another key factor, namely the possibility for personal and cultural development to emerge out of the dialogue process. Two public

participants who identified themselves at the start of the project as "likely opposers" reported appreciating diverse perspectives and the freedom to express their views—but also that their own views changed as a result of the openness of the dialogue. As an example, Public Member 1, from a Catholic background, described how their opinions evolved and their "mind was changed" by the exercise:

> When I first read it all at the beginning, I was probably against it because I was thinking, no, that's an embryo, what if it feels pain? What if it has a soul? No, I couldn't really agree with that. But how my mind was changed, information is power, isn't it?—Public Member 1

Whether or not their minds were changed, participants also valued their exposure to a range of views in terms of gaining deeper insights into the views of others. Public Member 3, whose views were shaped by living with a family member with a developmental condition as well as by their Christian faith, did not change their overall opinion but enjoyed gaining a better understanding of opposing views, for example through hearing personal stories from individuals who had had children through assisted reproduction:

> When you actually spoke to and heard stories from people and how the drive to have children really affects people, from that I can see a different side of what people are doing in terms of embryo research.—Public Member 3

Professional Member 3 noted that including religious perspectives challenged preconceived notions and improved methodological robustness.

INFORMAL COMMUNICATION, DOWNTIME, AND REFLECTION

In addition to content, interviewees in our study also drew attention to formal aspects of the dialogue exercise that were seen to contribute to its success. These included the amount and pacing of communication, both of which were seen to play an important role in enabling the kind of reflection that allowed participants to "see different sides," as mentioned earlier. Some participants emphasized the importance of informal conversation and reflection periods as a way to build relationships, enhance understanding, and incorporate additional viewpoints. Varying the format of communication also mattered. For example, Professional Member 1 mentioned the value of one-on-one conversations during coffee breaks at in-person workshops during which participants could ask clarifying questions, improving both their understanding of the topic and relationship-building within the group.

While these face-to-face experiences were absent from online sessions, the initial webinars were praised by interviewees for including pauses and downtime. Both public and professional dialogue participants emphasized the importance of including time to reflect in the webinar.

> I particularly liked the webinar because there was a lot of time to kind of digest the materials and be able to kind of reflect and have time to think.—Public Member 2

Access to multiple online options was another aspect of the webinar praised for enabling space to explore related themes as well as time to reflect on their importance. A supplementary resources platform that was frequently used by participants before and between sessions offered the opportunity to complete activities, review workshop recordings, access news articles, submit questions, and engage in discussion boards.

The duration of the dialogue exercise also allowed for discussion of topics outside of formal sessions, and this too was viewed as advantageous. In keeping with Sciencewise's guidelines, participants were neither discouraged nor encouraged to discuss dialogue topics with family and friends. Public Member 1 engaged their family in these conversations, which in turn was described as having influenced their own opinion about human embryo research—again reinforcing the theme of time to reflect, explore, and understand different sides of the issue, but also, in this case, further supporting the importance of casual discussion to the process of evaluating complex information.

Taken together, incorporating break times, reflection periods, access to supplementary resources, and allowing space for informal discussions outside the group over the course of the exercise were all identified as helpful components of the HDBI dialogue by public and professional members. These elements were valued as means to increase understanding, improve relationships, reflect on "different sides", and "test" ideas, to arrive at more informed, nuanced, and confident views on a highly technical topic that many of the public participants had never previously encountered and knew little about.

HIGH-QUALITY FACILITATION

The role of facilitators in delivering a successful dialogue and engagement experience for public and professional participants is clearly a paramount concern in exercises of this kind. Effective facilitation is crucial for fostering respectful, participant-focused, and informed but impartial discussion. Five of 11 interviewees explicitly highlighted the skill and experience of facilitators as a major contributor to the success of the HDBI dialogue.

In addition to logistical and practical guidance, facilitators also provided

emotional support and were praised for having handled emotionally charged topics well.

> The facilitators clearly had a lot of experience in dealing with these sorts of elusive personal aspects of the issues that may come up and I thought they handled it very well.—Professional Member 4

Facilitators also acted as moderators to help support and guide group discussion. This is a role for which people are trained, and the UK has a larger number of such professionals than most countries due to the importance of engagement and dialogue to UK science policy (Burchell et al., 2009). Professional participants, who relied on the facilitators to decide when to interject with factual information without overtaking the conversation, appreciated the assistance this offered to dialogue.

> I really relied on the facilitators to judge when to bring me in or not, because for the most part I didn't want to say anything because […] I think as an expert the risk is that you can overtake a conversation.—Professional Member 2

Facilitators also helped to create a safe and inclusive environment by inviting quieter participants to share their thoughts and tactfully preventing any individual group members from dominating discussion. They also moved the discussion forward and helped to cover as much ground as possible by raising questions, encouraging interaction, and integrating viewpoints from breakout groups. This role was appreciated by participants for several reasons, including the ability to hear and reflect on opposing points of view in a well-structured environment that enabled all voices to be heard.

> I would speak about my opinion for a bit and they would say "we're going to come back to you, but we're going to have to move on and get everybody's point of view" […] At the end we would have a chance to disagree or add to other people's thoughts or change our initial opinions […] You mightn't think of the other implications of what you've said until someone steps up and says "but hold on a minute have you thought about this"—Public Member 1

ONLINE AND IN-PERSON DIALOGUES

There is a limited literature on the pros and cons of online versus face-to-face engagement and dialogue, despite the significant increase in the use of online webinars in the post-COVID period. Views about the comparative value of online versus in-person dialogue were a common topic of discussion among participants, with interviewees identifying a range of benefits and limitations to both formats (see Table S2). Although in a preliminary manner, our findings pointed to several key themes in this area that could inform future research and dialogue project design. For example, while virtual dialogues are often perceived to cut venue and travel costs, Professional Members 5 and 8 questioned if they are truly cheaper, as expensive virtual tools are needed for high-quality, interactive experiences.

> Commissioners tend to think about online dialogue being cheaper and quicker. It's not really [either of these] because you have to design it just as carefully, if not more carefully, to make it work well online.—Professional Member 8

The strongest argument in favor of virtual dialogue was its accessibility. Professional Member 3 noted that online formats can make dialogues accessible to people with constraining home and work situations. This view was echoed by multiple interviewees.

> Having a virtual aspect to the sessions also enables different people to participate because there will be people who won't be able to physically come to sessions, who would be able to take part virtually.—Professional Member 4

Additionally, Public Members 1 and 2 stated that online dialogues felt less overwhelming. The online platform allowed them to consider their thoughts and opinions before speaking, reducing the initial intensity of being introduced to new concepts as well as new people in a face-to-face setting. Despite these benefits, interviewees highlighted several limitations of online sessions, including the lack of spontaneity and flow and the related need for more direct prompts from facilitators, whereas in-person dialogues allowed participants to converse more freely. At the same time, greater facilitation and less spontaneity, as well as the option of self-directed interaction with online materials, were also seen to have their own positive effects on success. Online discussions were praised for better management and fewer interruptions, with chat functions allowing participants to introduce new ideas without speaking over each other. Conversely, the relational benefits of more animated conversation in a live group setting were often reduced, or lost entirely, in online activities, and there was an accompanying concern that participants might be distracted by their home or work environments. Our findings suggest that the greatest benefit may lie in hybrid approaches, but that timing also matters. Less intimidating introductory sessions can enable participants to become comfortable with each other, with the benefits of socialization and flowing discussion in the in-person discursive workshops developing subsequently.

WHAT IS NEUTRALITY IN PUBLIC DIALOGUE?

A key objective of the HDBI dialogue project was to inform, explore, and develop participant viewpoints without imposing any particular consensus or bias. In general, public engagement and dialogue projects are neither designed to reach definitive conclusions nor to establish an "objectively neutral" middle ground. They are instead intended to create a context in which the exchange of views can be mutually illuminating to all involved *because* they avoid steering the group toward any foregone conclusion or determining the "right" answer. While interviewees generally agreed that the dialogue was successfully structured to avoid these outcomes, there were different views as to whether neutrality was achieved, or indeed, if it is ever truly possible. The HDBI dialogue featured a broad oversight group with representation from diverse fields including medical ethics, developmental biology, sociology, history, fertility advocacy, public dialogue, policy, medical history, and law. This diverse input combined with skilled facilitation was praised in retrospective assessments of the project by one participant as "a good mix":

> This was a very good oversight group, very engaged, very excited about the work [...] they came from a lot of different fields, a good mix, and they played a very active role in framing the dialogue, but also in contributing as specialists and then disseminating the report and sharing the findings more widely at the end.—Professional Member 8

Careful delivery of dialogue was crucial for this blend of perspectives to generate a lively and challenging exchange that could push participants to "test" their own viewpoints, and disagree, but also ask questions and become better informed. Such a format requires careful balances and a degree of restraint. It is important, for example, that scientific literacy is not a barrier to participation. Scientific specialists acknowledged this tension: they wanted to correct biological details in order to be "fully accurate" but also knew they needed to act primarily as observers. This approach challenged them but ensured participants remained central to the dialogue.

However, some participants felt that a complete representation of diverse views was not fully achieved. Public Member 3, who opposed embryo research, felt their views were acknowledged but given less weight compared to those in favor. Public Member 2 felt that the dialogue was not biased in any direction but noted that it contained limited voices opposing human embryo research or extension to the 14-day limit.

> What I would say is that it would have been good to hear more voices from people who may be against the extension [...] I didn't really hear that much in terms of [...] people who would have been in disagreement about the extension of the fourteen-day window.—Public Member 2

Despite these concerns, interviewees cited participants' development of independent ideas, such as reversible regulation and lay summaries of research, as evidence of freedom from bias.

> They had lots of opportunities to identify both the downsides and the potential benefits of the research and … how they wanted to see research governed in the future … they came up with a lot of their own suggestions, particularly on the governance side which weren't being proposed or suggested by the researchers at all.—Professional Member 8

From the interviews conducted, it thus appears that while the dialogue was perceived to be as inclusive and unconstrained as possible, achieving a full spectrum of views may be unfeasible given the small size of the group and the short time frame of the project. One professional member suggested that, even if the organizers genuinely had no agenda, participants may perceive one, although they added that they felt the public participants would still be confident in their own views. Another professional member noted that since the dialogue is shaped by both organizers and participants, who all bring their own lived experiences to bear on their evaluation of the questions at hand, nobody involved can be fully objective and neutral. This interplay helps balance any perceived agendas and might approximate a rough neutrality overall.

> My experience is that there was probably enough intransigence on both sides that perhaps something kind of averaged out in the middle.—Professional Member 3

DISCUSSION

Public consultation on human embryo research has continued to expand and evolve for over 40 years. Since 2000, the importance of public engagement and dialogue concerning innovative and controversial scientific developments has become official UK science policy. As a result, the UK has a comparatively well-developed public engagement and dialogue skill base, with several groups having emerged to provide this service, including some, such as Sciencewise, that are partially funded from within government. As methodologies for conducting dialogue and engagement exercises continue to evolve and means of evaluating such exercises also develop further, studies such as ours can help provide empirical evidence and insight into considerations and strategies for effective public

dialogue on sensitive topics such as human embryo research.

Our study looked at a novel dialogue exercise from the point of view of trying to identify factors that influenced public and professional participants' views of successful outcomes. Based on in-depth data collected from a subset of dialogue participants through interviews, we concluded that four key groups of criteria emerged as "priority factors" determining success in this particular case study. These are schematically outlined in Figure 2, and in our discussion, we have selectively illustrated these broad groupings using examples of insights gained from participants. As these examples show, the ability for participants to learn, engage, and increase their own understanding of a complex topic benefited from a number of specific dialogue features. In addition to skilled facilitation, participants valued a mixed set of opportunities to improve their understandings and refine their views. These included the value of face-to-face interaction combined with independent learning; the benefits of online independent and group work combined with live, in person sessions; and the ability to hear from professional specialists dealing largely in facts and also from other members of the public who expressed different viewpoints. We also found that good facilitation, time to reflect, and casual conversations with family and friends provided important opportunities to explore, test, refine, and change their views. To ensure a "good mix" of views and materials, the HDBI dialogue provided multiple resources and opportunities to participants that included reasons to oppose as well as support extension of the 14-day limit and/or human embryo research in general. These included discussion prompts, pre-recorded videos, and specialists who presented arguments for opposition. This model of constructive communication, informed debate, and friendly curiosity ensured a dialogue that was robust and reflective of societal values, providing a hopeful example for building relationships among different communities. Future dialogues could learn from models like the AAAS Dialogue on Science, Ethics, and Religion, which focuses on relationship-building between scientists and faith communities, engaging with experts on how to navigate the historical events that have built such tension and training facilitators to converse about complex topics such as the pro-life movement (AAAS, 2020; O'Malley et al., 2021).

Public dialogue is integral to many research fields, such as mitochondrial donation, germline gene editing, and artificial intelligence. In germline gene editing, clear goals for public engagement have been identified, including fostering deliberation on "what-if" scenarios, exploring value-based agreements and disagreements, involving diverse perspectives, and informing socially aligned policymaking (Geuverink et al., 2024). In common with human embryo research, literature from germline gene editing emphasizes the need to embed public dialogue into the scientific research infrastructure to enhance its credibility (Scheufele et al., 2021). In the field of AI, ethical concerns echo those in human embryo research, particularly around respect for humanity, unforeseen future consequences, and informed consent (Pickering, 2021). Challenges to public engagement include how to navigate the complexities and uncertainties of the topics, promote transparency, counter misinformation, and facilitate inclusive dialogue (Adams and Burall, 2019). A systematic review of public dialogues on ethically contentious subjects could further clarify how generalizable findings are across fields and help define what methodologies are most effective at facilitating dialogue on such topics.

ACKNOWLEDGMENTS

We thank the individuals who participated in our study. We are grateful to Naomi Clements-Brod and Michael Norman for their comments on the manuscript. P.J.R.-G.'s group is supported by grants from the BBSRC (BBS/E/B/000C0522), MRC (MR/T011769/1 and MR/V02969X/1), and Wellcome (215116/Z/18/Z and 225839/Z/22/Z).

AUTHOR CONTRIBUTIONS

Conceptualization, S.F. and P.J.R.-G.; methodology, M.B., S.F., and P.J.R.-G.; investigation, M.B.; data curation, M.B.; analysis, M.B.; writing, M.B., S.F., and P.J.R.-G.; project administration, P.J.R.-G.; funding acquisition, S.F. and P.J.R.-G.; supervision, S.F. and P.J.R.-G.

DECLARATION OF INTERESTS

The authors declare no competing interests.

SUPPLEMENTAL INFORMATION

Supplemental information can be found online at https://doi.org/10.1016/j.stemcr.2025.102498.

REFERENCES

AAAS (2020). Dialogue on Science, Ethics, and Religion. https://www.aaas.org/programs/dialogue-science-ethics-and-religion.

Adams, L., and Burall, S. (2019). How to stimulate effective public engagement on the ethics of artificial intelligence. https://www.involve.org.uk/sites/default/files/uploads/docuemnt/How%20to%20stimulate%20effective%20public%20debate%20on%20the%20ethics%20of%20artificial%20intelligence%20_0.pdf.

Burchell, K., Franklin, S., and Holden, K., (2009). Public Culture as Professional Science: Final report of the ScoPE project (Scientists on public engagement: from communication to deliberation?). London School of Economics BIOS Centre. https://core.ac.uk/download/pdf/1540092.pdf.

Franklin, S., and Jackson, E. (2024). The 14 Day Rule and Human Embryo Research (Routledge).

Geuverink, W.P., Houtman, D., Retel Helmrich, I.R.A., van Baalen, S., van Beers, B.C.,

van El, C.G., Henneman, L., Kasprzak, M. D., Arets, D., and Riedijk, S.R. (2024). The need to set explicit goals for human germline gene editing public dialogues. J. Community Genet. *15*, 259–265. https://doi.org/10.1007/s12687-024-00710-1.

Hopkins, H., Kinsella, S., Ingram, H., and Hearing, J. (2023). Public Dialogue on Research Involving Early Human Embryos. Zenodo. https://doi.org/10.5281/zenodo.14926481.

Hyun, I., Wilkerson, A., and Johnston, J. (2016). Embryology policy: Revisit the 14-day rule. Nature *533*, 169–171. https://doi.org/10.1038/533169a.

Lovell-Badge, R., Anthony, E., Barker, R.A., Bubela, T., Brivanlou, A.H., Carpenter, M., Charo, R.A., Clark, A., Clayton, E., Cong, Y., et al. (2021). ISSCR Guidelines for Stem Cell Research and Clinical Translation: The 2021 update. Stem Cell Rep. *16*, 1398–1408. https://doi.org/10.1016/j.stemcr.2021.05.012.

Nisbet, M.C., and Scheufele, D.A. (2009). What's next for science communication? Promising directions and lingering distractions. Am. J. Bot. *96*, 1767–1778. https://doi.org/10.3732/ajb.0900041.

O'Malley, R.C., Slattery, J.P., Baxter, C.L., and Hinman, K. (2021). Science engagement with faith communities: respecting identity, culture and worldview. J. Sci. Commun. *20*, C11. https://doi.org/10.22323/2.20010311.

Pickering, B. (2021). Trust, but Verify: Informed Consent, AI Technologies, and Public Health Emergencies. Future Internet *13*, 132.

Scheufele, D.A., Krause, N.M., Freiling, I., and Brossard, D. (2021). What we know about effective public engagement on CRISPR and beyond. Proc. Natl. Acad. Sci. USA *118*, e2004835117. https://doi.org/10.1073/pnas.2004835117.

Sugarman, J., Clark, A., Fishkin, J., Kato, K., McCormack, K., Munsie, M., Peluso, M.J., René, N., and Solomon, S.L. (2023). Critical considerations for public engagement in stem cell-related research. Stem Cell Rep. *18*, 420–426. https://doi.org/10.1016/j.stemcr.2023.01.002.

Yui, H., Muto, K., Yashiro, Y., Watanabe, S., Kiya, Y., Fujisawa, K., Harada, K., Inoue, Y., and Yamagata, Z. (2023). Survey of Japanese researchers and the public regarding the culture of human embryos in vitro beyond 14 days. Stem Cell Rep. *18*, 799–806. https://doi.org/10.1016/j.stemcr.2023.02.005.

Yui, H., Yashiro, Y., Muto, K., Watanabe, S., Kiya, Y., Inoue, Y., and Yamagata, Z. (2024). Opinions on research involving human embryo models by researchers and the general public. Regen. Ther. *26*, 9–13. https://doi.org/10.1016/j.reth.2024.05.001.

Stem Cell Reports, Volume *20*

Supplemental Information

Identifying enabling strategies for effective public dialogue in human embryo research

Matilda Beckett, Sarah Franklin, and Peter J. Rugg-Gunn

Table S1. Definitions of success for public dialogue, related to Figure 2.

Interviewee	Definition of success
**Public Member 1**	Create rigorous discussions that include diverse viewpoints without forcing consensus. Collect public perspectives and feed them into policy or practice.
**Public Member 2**	Ensure individuals leave with a more open mind and increased knowledge, with opportunities to provide personal feedback.
**Public Member 3**	Enable organisers and participants to listen to and engage with a diverse range of life experiences and viewpoints.
**Professional Member 1**	Focus on making sure everyone involved feels heard and able to express their opinions.
**Professional Member 2**	Capture public discussions to crystallise opinions and attitudes that may inform policy.
**Professional Member 3**	Catalyse changes in practice and of culture.
**Professional Member 4**	Collect findings that accurately represent public views and can be used for policy development. Foster positive relationships between stakeholder groups.
**Professional Member 5**	Ensure participants feel more engaged with the topic, and collect findings useful for policy, practice, or future dialogues.
**Professional Member 6**	Advance the field of public dialogue. Enhance participants' scientific knowledge and understanding of alternative viewpoints. Provide commissioners with insights into public perspectives.
**Professional Member 7**	Facilitate meaningful conversations between participants and scientists, and accurately capture these discussions.
**Professional Member 8**	Achieve pre-set objectives of design, policy impact and contribution to the professional field of public dialogue. Effectively disseminate findings.

Table S2. Comparison of Online and In-Person Public Dialogue, related to section on 'Online and in-person dialogues'.

	In-person dialogue	Online dialogue
**Cost**	Higher costs due to venue and travel expenses.	No venue or travel costs.
		Creating engaging online experiences can be costly.
**Accessibility**	Accessibility and convenience limited by geography, work hours, children, disabilities, carer duties, etc.	Allows participation from various locations with reduced travel and timing constraints. Online environment may be less overwhelming for some participants.
**Discussion and debate**	Stronger rapport through face-to-face interactions. Immediate answers to questions available. Increased social bonding and casual interaction.	Participants may feel less social pressure and provide more honest, unfiltered opinions.
	Potential for social pressure to reduce the openness of viewpoints expressed.	Harder to build rapport and relational goals. Discussions may be less memorable over time.
**Facilitation**	Easier to facilitate discussions when able to see body language. Increased spontaneity of discussion between participants.	Easier to manage speaking order and reduce interruptions. Chat function allows for non-interruptive additional points.
	Harder to manage interruptions and side-conversations.	Conversations tended to flow between facilitator and participant rather than between participants. Harder to respond to participants' non-verbal cues.
**External distractions**	Reduced likelihood of distractions due to being in a dialogue-specific location.	Higher risk of participants being distracted by events in their home or at work.

Note S1 – A summary of the HDBI Public Dialogue, related to the introduction.

The HDBI Public Dialogue project sought to engage a diverse group of the UK public to deliberate on early human embryo research with a specific focus on the 14-day rule. Co-funded and commissioned by the Human Developmental Biology Initiative and UK Research and Innovation Sciencewise, and delivered by Hopkins Van Mil, this foundational project aimed to hear and discuss public hopes and concerns for this area of research. This was a qualitative study to understand why views are held and what is important to individuals.

The project used targeted recruitment to obtain a range of levels of awareness of early human embryo research and regulation, and a mix of those who support and oppose this research, enrolling 70 members of the public from across the UK. To support the project delivery team, an Oversight Group was formed of 23 members comprising biologists and social scientists, third sector organisations and those involved in embryo research policy. Over the course of the project, 28 specialists provided input that covered biological sciences (12 individuals), regulation and legislation (4), philosophy and ethics (7), medicine (2) and patient views (3).

The project started with a pilot group of 9 members of the public, followed by a second phase of the project with 19 individuals who have lived experiences of developmental conditions, fertility treatment, or recurrent miscarriage. The process for the pilot and lived-experiences groups involved a webinar and three online workshops. The next stage of the project was with two general groups, each of 21 members of the public. For these groups, the last two workshops were held in-person. Presentations from specialists, lived experience films and infographics were used as stimulus materials for the discussions. An online platform, available between sessions, enabled participants to complete activities, watch workshop recordings, access news articles, submit questions, and participate in discussion boards.

Public participants of the project showed high engagement and fascination about early human embryo research. The current knowledge gaps in embryo research were easily grasped, particularly the limited information available on how embryos develop between day 7 and day 28. Ethical, moral and religious considerations featured throughout the discussions. There was a high level of confidence in the current regulatory and legislative structures that surround early human embryo research. Many participants voiced hope for further research particularly where there is opportunity for improvement in human health.

Participants' concerns for the future included worries related to the detection of certain health conditions or disabilities, which could then lead to the elimination of those conditions and without society's consent. There were also worries about genetic engineering, and whether embryo research would enable the creation of 'perfect' individuals. Concerns were raised about who was funding embryo research, and the possibility of inequitable access to future treatments.

Many participants supported some form of extension to the 14-day rule provided it aligns with societal expectations regarding respect for the embryo and the research remains rigorously regulated. Common reasons provided why individuals would support an extension to the current limit included the potential of the research to lead to improvements in IVF success rates, reduce the incidence of miscarriage, and to better understand, treat or prevent serious health conditions. Views differed between individuals on how the 14-day rule should change. Some believed the change should occur gradually, in small increments, with regular reviews. Others argued that extending the limit to 28 days should be considered, given the potential new benefits the research could offer, particularly during the 14- to 28-day developmental period. There was a strong interest to involve the public in future decision making on this area of research and that these processes are transparent.

An independent evaluation report concluded that the Public Dialogue project was well-designed, efficiently delivered, and successfully met all of the original objectives. The evaluation noted that conditions were successfully created to allow participants to share views and discuss sensitive topics. The mix of stimulus materials, ample time for small group deliberation both online and in person, and substantial involvement of specialists contributed to the effective delivery and impact of the project.

Note S2 – Participant recruitment and interviews, related to the section on ‘Defining successful public dialogue concerning human embryo research’.

Approval to conduct the interview-based research was obtained from the University of Cambridge Sociology Ethics and Risk Assessment for Research Committee. Eleven individuals who participated in the HDBI dialogue were interviewed. These individuals represent a broad range of positions within the dialogue: members of the public who participated in the exercise, professionals who oversaw design and delivery of the project, specialists who provided sessions for the participants during the exercise, and project evaluators. Interviews were conducted either in-person or online, and were semi-structured with an interview guide. Questions explored participants' experiences with the HDBI public dialogue, their views on different methodologies, and their personal definitions of success for public dialogues, among other topics. Interview recordings were transcribed using Microsoft Word Speech to Text and edited for accuracy. All identifying information was removed from transcripts and audio files were deleted after use. Analysis was indicative, formed by close reading and notetaking.

1: Contact message

Dear [Recipient's Name],

I hope this email finds you well. My name is Matilda Beckett, and I am reaching out to you regarding a study on high-quality public dialogue in early human embryo research. Your association with the HDBI public dialogue on this topic makes your perspective invaluable to our research, and I would like to invite you to participate in a 30-minute to 1-hour interview.

The purpose of my study is to gain insights into what constitutes high-quality public dialogue in early human embryo research and to explore why it matters. Your unique experiences and expertise would contribute significantly to the depth and richness of the findings.

Interviews can be conducted either online at a time convenient for you or, if you are based in Cambridge, in person. I understand the importance of your time, and I assure you that the interview will be structured efficiently while allowing time and space to explore interesting themes.

Your participation will contribute to advancing our understanding of public dialogue in this critical field.

If you are willing to participate, please reach out to me at [redacted].

I appreciate your time and consideration in contributing to this research endeavour.

Warm regards,

Matilda Beckett

MPhil Reproduction and Embryogenesis

University of Cambridge

2: Consent Form

Title of Project: What does High Quality Public Dialogue in Early Human Embryo Research Look Like and Why does it Matter?

Researcher: Matilda Beckett – MPhil Reproduction and Embryogenesis

About the Project: This project seeks to investigate the use of dialogue-based methods to understand public opinions on human embryo research. The project utilises the recent HDBI Public Dialogue on Early Human Embryo Research as a case study. By interviewing scientists, public engagement professionals and participants of the dialogue, the project aims to identify which aspects of public dialogue-based methods are most effective and which should or should not be taken forward to future engagement activities. The project also hopes to factor find by uncovering factors that influenced decision making for participants and researchers throughout the public dialogue process. The expected outcomes are i) a deepened understanding of what high-quality public engagement might look like for the field of early human embryo research, ii) a rationale as to why the dialogue aspect of public engagement is important and iii) anticipation of future directions for dialogue involving early human embryo research and similar topics.

During the interview you will be asked questions about your experience of the HDBI public dialogue activity, your role in the public dialogue and hypothetical questions about the future of public dialogue. Participation is voluntary and you have the right to withdraw at any time with no consequence by contacting the email provided on this form. Interviews will be audio recorded on a secure device. Personal identifying information will be removed, and transcripts will be analysed for overall themes. Some quotes may be used in the project write-up. Interviews will be carried out in-person and online. Some interviews may also be carried out using questionnaires.

Your data will be processed according to the guidance provided by the University of Cambridge regulations on secure storage and use of participants' data. Anonymised transcripts will not be made available for use by researchers other than those within my supervisors' groups at the University of Cambridge and the Babraham Institute. You may consent or refuse consent to future use of interview data below.

If you would like more information about the data storage and participant anonymisation methodology for this project, please feel free to contact me at [redacted].

The University's regulations on the secure storage and use of participants' data may be found at https://www.information-compliance.admin.cam.ac.uk/dataprotection/research-participant-data

1. I confirm that I have understood the purpose of the project and my participation in it, and I have had the opportunity to ask questions. ☐

2. I understand that my participation is voluntary and that I am free to withdraw at any time without giving any reason. ☐

3. I understand that my responses will be anonymised and used only for academic research. ☐

4. I understand that my interview may be recorded. ☐

5. I agree to take part in the above project. ☐

6. I consent for anonymised data collected through these interviews to be stored securely and potentially used in follow up studies. Yes ☐ No☐

Signatures

Participant name signature and date

Researcher name signature and date

3: Interview Guide

Key	
**Aim to ask to everyone**	*
**Of specific importance to professional members**	**
**Of specific importance to public members**	***

Can you tell me what your role was in this public dialogue project and how you got involved?	*
What was your experience of public dialogue before this?	*
How would you define public dialogue? What is your understanding of the difference between public dialogue and public outreach?	*
What activities and processes did participants/you go through in the HDBI public dialogue?	*
How can "success" be defined for a public dialogue activity?	*
Are there any aspects of public dialogue that you think make the dialogue activity more or less successful?	*
Do you think the HDBI Public Dialogue on Early Human Embryo Research was a success?	*
What are some examples of what went well in the HDBI public dialogue? Was this related to any particular people or activities?	*
What are some examples of aspects that either didn’t go well or could be improved?	*
How were discussions between participant groups facilitated?	*
Do any particular workshops, speakers, or activities stand out to you as particularly vital, decisive, or influential?	*
One of the aims of public dialogue is to encourage participants to come to conclusions unencumbered by the conscious/unconscious goals of the project researchers. Do you feel like this worked?	*
Considering specifically the aspects of the dialogue relating to stem cell-based embryo models, were there any particular strengths or limitations to this part of the dialogue which come to mind?	*

Do you think that in future dialogues it would be possible to run dialogues completely remotely? Were there any benefits to online versus in-person sessions?	*
I’ve experienced opinions in my cohort that the public is not interested in embryo research and is not well-informed enough to be involved. What would your response to this opinion be?	*
During the HDBI dialogue, did there appear to be any "tipping points" for participants/you changing or steadying opinions? What were they? Were these associated with any tasks or speakers?	*
In terms of translating public dialogue results into policy implications, how do you envision this going forward?	*
Was there anything that really surprised you during the public dialogue process, anything you weren’t necessarily expecting?	*
Is there anything else that you think I might want to know that I’ve not directly asked about?	*
Considering the different groups of participants from the northern, southern, and lived experience groups, did you observe any differences in the flexibility of their opinions, or did they all seem equally plastic?	**
Do you think it makes sense to have a separate lived experience group? If so, why?	**
What do you feel are the main limitations of public dialogue overall?	**
Did you come into the dialogue feeling like you already knew what your opinions would be? Do you think other participants had the same/different levels of plasticity?	***
Now that you have been involved in two relatively lengthy public dialogues, would you still consider yourself a layperson on the topic of human embryo research?	***
